# Five years of experience teaching pathology to dental students using the WebMicroscope

**DOI:** 10.1186/1746-1596-6-S1-S13

**Published:** 2011-03-30

**Authors:** Janusz Szymas, Mikael Lundin

**Affiliations:** 1 Department of Clinical Pathology, University of Medical Sciences. Przybyszewski St. 49. 60-355 Poznan, Poland; 2 Institute for Molecular Medicine, Finland FIMM, P.O. Box 20, FN-00014, University of Helsinki, Finland

## Abstract

**Background:**

We describe development and evaluation of the user-friendly web based virtual microscopy - WebMicroscope for teaching and learning dental students basic and oral pathology. Traditional students microscopes were replaced by computer workstations.

**Methods:**

The transition of the basic and oral pathology courses from light to virtual microscopy has been completed gradually over a five-year period. A pilot study was conducted in academic year 2005/2006 to estimate the feasibility of integrating virtual microscopy into a traditional light microscopy-based pathology course. The entire training set of glass slides was subsequently converted to virtual slides and placed on the WebMicroscope server. Giving access to fully digitized slides on the web with a browser and a viewer plug-in, the computer has become a perfect companion of the student.

**Results:**

The study material consists now of over 400 fully digitized slides which covering 15 entities in basic and systemic pathology and 15 entities in oral pathology. Digitized slides are linked with still macro- and microscopic images, organized with clinical information into virtual cases and supplemented with text files, syllabus, PowerPoint presentations and animations on the web, serving additionally as material for individual studies. After their examinations, the students rated the use of the software, quality of the images, the ease of handling the images, and the effective use of virtual slides during the laboratory practicals. Responses were evaluated on a standardized scale. Because of the positive opinions and support from the students, the satisfaction surveys had shown a progressive improvement over the past 5 years. The WebMicroscope as a didactic tool for laboratory practicals was rated over 8 on a 1-10 scale for basic and systemic pathology and 9/10 for oral pathology especially as various students’ suggestions were implemented. Overall, the quality of the images was rated as very good.

**Conclusions:**

An overwhelming majority of our students regarded a possibility of using virtual slides at their convenience as highly desirable. Our students and faculty consider the use of the virtual microscope for the study of basic as well as oral pathology as a significant improvement over the light microscope.

## Background

There is an increasing tendency at medical universities to digitize whole microscope histopathological slides from teaching collections for web-based studies [1]. Virtual microscope - WebMicroscope using digitized slides as Enhanced Compression Wavelet (ecw) file, format pioneered in aerial and satellite imagery, offer an alternative to the other web-based method of teaching pathology [2]. It makes microscope laboratory studies in pathology more efficient. This expands a traditional microscopic study allowing the student to examine entire tissue sections. It is also opposed to static images of selected fields. In this way, digitized slides can be visualized at any magnification and moved in the x-y axis. This nearly perfectly emulates a traditional microscope and a glass slide [3]. Virtual microscopy allows students to independently explore the entire histologic slide. Abandoning the use of conventional training microscopes and glass slides, we decided to rely on virtual microscopy to facilitate learning of pathology and to radically redesign laboratory practicals for teaching basic and oral pathology to dental students. This article describes 5 years of our experience with this experiment and some of the surprises we met in the evaluation by the students and staff.

## Methods

### Technical details

We began working with the WebMicroscope at the Department of Pathology, the University of Medical Sciences in Poznan, Poland, in 2005 as we adapted our research-based technology of robotized microscopy for education. Glass slides were digitized using the Axioskop 2e microscope with a motorized stage, autofocus, digital high resolution color camera, and a PentiumV computer with four gigabytes of RAM and Windows XP Pro, all coordinated by Axiovision (Zeiss) software. Up to 2500 contiguous 200x or 400x high power microscopic fields with various resolution of digital camera (Table 1) were automatically captured and tiled together into a seamless montage. Conceptually, the digitized slide format is a pyramidal stack of compressed images with a full size, full resolution image at the base and a small, low resolution image at the top. A zoom function jumps from one layer in the stack to the next, changing magnification by four fold with each click of the mouse. The click and drag function allows scanning each layer of the stack in the x-y axis. The WebMicroscope server software delivers the files from the web server on demand, and virtual slides are displayed in standard HTML frames on a Windows platform with the WebMicroscope plug-in. During the course, all virtual slides used for laboratory practicals can be viewed at http://ampat1.amu.edu.pl/ that is a public domain site. Currently, only Windows platform is adequately supported. We are planning to support all platforms in academic year 2010/2011.

**Table 1:** Available scaling for Axioplan 2e microscope as a virtual slide generator

Objective	Resolution of digital camera	X-Scaling μm/pixel	Y-Scaling μm/pixel
20x (1300 x 1030 pixels)		0.53 μm	0.53 μm
40x (1300 x 1030 pixels)		0.26 μm	0.26 μm
20x (2600 x 2060 pixels)		0.25 μm	0.25 μm
20x (3900 x 3090 pixels)		0.17 μm	0.17 μm
40x (2600 x 2060 pixels)		0.13 μm	0.13 μm
40x (3900 x 3090 pixels)		0.08 μm	0.08 μm

### Implementation in basic and systemic pathology

In the fall semester of 2005, we carried out a formative evaluation of the WebMicroscopy laboratory practical for the third-year dental students course but only for one student group (8 students). Glass slides from previous unit of the laboratory practicals were digitized, placed on the WebMicroscope server and made available to the students on the internet. During the first laboratory practical, traditional student microscopes and glass slides were also available as a supplement. At the end of the first laboratory practical, students rated the Virtual Microscope as superior to the traditional microscope in terms of clarity of morphologic images, the ease of use, efficiency and accessibility. Considering this positive evaluation, subsequent practicals were conducted using exclusively the WebMicroscope. A set of over 100 histopathological slides was subsequently digitized and used in the 2005 fall semester (basic and systemic pathology) and 2006 spring semester (oral pathology). In this way, the virtual microscope has become the only device used by those students during laboratory practicals. Whereas this “experimental” group was taught with the WebMicroscopy in a computer lab with two students per computer during the two introductory semesters, remaining students were taught in a traditional laboratory. An assistant or adjunct was present in all sites to interact with the students at individual workstations or microscopes.

### Implementation in oral pathology

In the second semester of third year course, students have 15 laboratory practicals. Students examine microscopic slides and associated patient clinical data in small groups during supervised laboratory practicals in preparation for examination. At the end of such a course, there is an obligatory practical exam. For the spring 2006 Oral Pathology laboratory practicals, all the 50 slides previously unit of the laboratory practicals were digitized and placed on the web. Digitized slides were linked with macro images, radiological images, and patient case histories, all of which had been put on the web systematically since 1993 [4]. Thus, the only variable in 2006 was adding the virtual slides to the web.

### Criteria and methodology

Considering very good subjective as well as objective evaluation by our first “experimental” group in academic year 2005/2006, we have decided to go further and prove the usefulness of digitized slides to all students in the next years. Therefore, since academic year 2006/2007 we switched from traditional pathology microscope practicals to WebMicroscope-driven laboratory practicals in our third-year pathology course for all dental students. Additionally, we have experienced a growing number of students - from 64 in year 2005 to 116 in 2010. Instead of preparing additional glass slides for new students we have concentrated on completing and exchanging the existent glass slides with new cases. Not only all the slides used for laboratory practicals were completely digitized but we also added and/or replaced approximately 10 - 20 digitized slides every year. Virtual microscope WebMicroscope) was the only device used by the students during laboratory practicals conducted in up to 9 student groups. The laboratory practicals were supervised by staff and attendance was mandatory for the students. The evaluation system was based on credits obtained by the students during practicals and final examinations. In oral pathology, passing the practical exam was necessary to validate. Feedback from students was performed after the final examination.

## Results

### Students evaluation and staff opinion

We undertook a comprehensive evaluation of the implementation of virtual microscopy at the end of every academic year. A questionnaire was distributed to all students who were asked to provide ratings from 1 (low) to 10 (high) for effectiveness, image quality, ease of use and usefulness of virtual slides. They were also asked to rate specifically the usefulness of practical laboratories in basic, systemic and oral pathology. In addition, space was provided for comments.

Altogether, we collected data from 365 of the 412 students in years 2006 - 2010 with response rates of 98% to specific questions requiring a rating. Overall, the evaluation was overwhelmingly positive. The WebMicroscope as a didactic tool during laboratory practicals was rated over 8/10 in 2005-2010 in basic and systemic pathology and has risen to 9/10 in oral pathology on a 1-10 scale as various student suggestions were implemented. Students rated the effectiveness of both virtual slides and the viewer software very highly (Table 2). Of particular interest was a very strong support for the integration of clinical and pathological data. Not all students offered free-response comments. We coded the comments as either positive or negative. There were 255 positive and only 15 negative comments. Frequent comments were: “The WebMicroscope software is great…” , “You can open it everywhere…” , “I hope that other departments will follow the Professor…” , “I think that the WebMicroscope is a great educating tool”, “It’s much easier to learn if everybody can analyze the specimens themselves on a WebMicroscope and not struggle with glass slides”, “A very convenient and efficient way to practice pathology…”, “I think that this software is very useful and interesting…”, “Interesting and easy, I will memorize some of the specimens for sure…”. Over the years, a number of students had made negative comments to staff members about the digitized slides, especially for basic and systemic pathology. Negative comments concerned technical issues. However, those statements were usually not entirely critical: „Some problems to open the first specimen. You need to download the right software…”, „The WebMicroscope browser opens slowly …”, „No detailed descriptions of specimens on the site with the microscope”, „There should be some macroscopic specimens (photos of mucosal lesions) …”, „No oxygen in the room of Coll. Stomatologicum”, „Too bad there’s no air-conditioning in the computer room.”, „Horrible organization of the tests – long waiting..”, „Sometimes the assistant „kept a distance”, did not put much work to prepare himself for the practical”. We were therefore surprised by the strength of appreciation for the WebMicroscopy that was revealed by the questionnaires (Table 2).

**Table 2:** Usefulness of WebMicroscope was rated in:

2005/2006 n = 8 students	2006/2007 n = 84 students	2007/2008 n = 92 students	2008/2009 n = 102 students	2009/2010 n = 116 students
basic and systemic and oral pathology mean = 9.4 ± 0.9	basic and systemic pathology mean = 8.2 ± 1,9	basic and systemic pathology mean = 7.9 ± 1.9	basic and systemic pathology mean = 8.4 ± 2.4	basic and systemic pathology mean = 7.8 ± 2.3
	
	oral pathology mean = 9.0 ± 1.5	oral pathology mean = 8.2 ± 1.7	oral pathology mean = 8.7 ± 2.3	oral pathology mean = 8.8 ± 1.6

The staff valued the efficiency of the use of the WebMicroscope at the Department of Clinical Pathology, University of Medical Sciences in Poznan. This approach has allowed to analyze 8 – 12 slides per 2-hr class, with a wide-range discussion. Staff members were pleasantly surprised by the striking evidence of active and independent learning within practical classes, as well as the extent to which the students were willing to undertake collaborative group work. Moreover, many students asked questions seeking further details about pathological changes so that they could understand the relationship of morphological changes to the clinical manifestations of the disease. It was equally gratifying to see how well our students solved problems in understanding histopathological images when they learnt the relevant clinical data. In class, they often asked in-depth and thoughtful questions about how abnormalities that they identified developed.

### Benefits of use of WebMicroscope

Although the major innovative aspect of virtual slide technology is that the computer workstation can emulate a traditional student microscope and glass slides, there are several other educational advantages of digitized slides. A major advantage is the efficiency and accessibility. The efficiency is due to the fact that students have all the slides accessible at a click at anytime and anywhere, in focus, with proper lighting and condenser adjustment, and a far superior quality in comparison with common quality of student light microscope. Slides are also annotated and integrated on-line with patient history, macro and radiological images, and linked with other web-based resources. This presents a markedly different experience from sitting at a traditional microscope with a photocopied paper data. Because the virtual slides are always in focus and with proper lighting, students can concentrate all their efforts on the content of the slide rather than the technical aspects of using a traditional microscope. Also, the single best slide from the existing collection or one available glass slide can be digitized and made available to all students at the same time. The interactions between students are enhanced. Several students can look at the same virtual slide image on a monitor and compare with their neighbors at an adjacent workstation, whereas a traditional laboratory may have predominantly single-headed microscopes. Already during the first laboratory practical held in the computer laboratory we observed a marked increase in the discussions among students as compared to the traditional practical.

As anticipated from the evaluation, students preferred the WebMicroscope-based laboratory practicals. The majority of students attended the scheduled practicals but many preferred additional access to the WebMicroscopy on their own outside the scheduled hours. Server log files indicated ca 8000 - 9000 home page hits per year.

The faculty benefit from virtual slide technology in that they can prepare, in their offices, for teaching in lab practicals and discussion groups, design virtual slides in lecture hall and laboratories, and can better interact with students as they observe virtual slides on the computer screen. The faculty can also easily download screen shots of virtual slides for use in other computer-based educational programs. The institution benefits because, as microscope laboratories are moved into computer rooms, will not have to support both computer laboratories and large expensive microscope laboratories.

## Discussion

In the context of standardize curriculum and a growing number of students, various strategies have been employed to improve the student experience of learning pathology. These have ranged from the use of digitized still images delivered via the Web [5,6] to the restructuring of class and schedules formats given to lectures versus laboratory teaching [7]. Because even well annotated on-line atlases of static images simply cannot substitute for examining a slide in terms of learning microscopic pathology, the most significant technological innovation has been the introduction of virtual slides [8,9]. While different medical schools have adopted virtual slides either gradually [10] or precipitously [11], the future of virtual slides is assured, especially with steady improvements in scanning and display technologies as well as the development of teaching collections such as those at the University of Iowa or Medical University of South Carolina, Charleston, USA [12,13]. In previous report, Kumar and colleagues [14] had demonstrated that because of their significant benefits to the user, the Web was in fact preferred by many students who never achieve technical competence with a classical microscope. In our approach, we have taken the application of virtual microscopy considerably further, completely abandoning the use of glass slides and integrating the virtual slides into teaching of basic and oral pathology. We were delighted that students gave it an enthusiastic endorsement. The success of the experiment was mainly due to the excellent service provided by the University of Helsinki and the IT Center of the University of Poznan. Although various minor technical difficulties did arise periodically, they were dealt with promptly and there were no significant disruptions of the teaching sessions during 2005 – 2010. For whole slide imaging we are using standard high quality automated light microscope equipped with scanning stage and digital camera. This technology of raster scanning nowadays is used in plenty of digital slides scanner e.g. VS-110 (Olympus), Mirax (Zeiss), Pannoramic Scan 150 (3DHistech), Metafer V-Slide (Metasystems). Slides constructed on this manner are of the highest quality and mostly awarded. Using standard microscope instead encapsulated scanner for us is economically consider the number of slides scanned in every year for didactic purposes. One of the major points emerging from the evaluation data was the importance of providing an appropriate context for learning. Dental students clearly perceived benefit from studying basic pathology and oral pathology in relation to case studies and the relevant histopathology with the use of the WebMicroscope. Such an approach is likely to be advantageous for learning in both pathology and clinics in comparison to sequential courses. Our previous experience with a program based on traditional teaching methods was that despite some cross-disciplinary efforts over the years, students often did not achieve a good understanding of pathology in their third year of study and they frequently exhibited exasperatingly little knowledge of histopathology during clinical classes in years 4 and 5. We believe that teaching pathology based on digitized cases viewed on a virtual microscope had important benefits for students and helped to remove the boundaries between disciplines. Moreover, with the use of virtual slides, the staff felt that their time was used more effectively because the questions from students generally concerned the pathological changes themselves rather than technical problems or inability to find the relevant areas of the slide. We believe that an integrated approach was considerably facilitated by the availability of virtual slides for teaching and learning. Virtual microscopy offers other benefits, especially by helping to build a community of students. Overall, we believe that this novel approach has been a success. We wish to emphasize that student learning with virtual slides does not terminate after the practical exam. We do believe that histopathology teaching will continue through the fourth and fifth year of the 5-year program as part of virtual cases or PBL. We have already expanded the virtual slide collection, incorporating new diseases, various microscopical techniques, special stains and molecular methods in histopathology. To date, we have taken advantage of the possibilities offered by the virtual slide software to annotate specific and selected fields at appropriate magnifications. However, this is not all that is on the agenda of future improvements [15,16].

## Conclusions

We use the best currently available didactic tool - whole slide imaging (WSI), in order to prepare students to enter the mysterious world of diseased human cells and to succeed at their medical practice. Dental students have not only accepted this technology but have also shown enthusiasm for the development of further on-line resources for learning pathology. Our students now believe that working on their pathology skills using virtual microscopy will make them more knowledgeable and competitive in clinic, research, and dental practice. We believe this new cutting-edge virtual slide technology has also a significant potential for innovation beyond medical student and undergraduate education. This includes web-based publishing of atlases, textbooks and articles, continuing medical education, pathology resident education, learning / exploring the pathology of experimentally manipulated animals, proficiency testing, and certifying examinations.

Although the initial equipment and software cost for creating virtual slides is high, we believe that this new technology has a potential to revolutionize the way we teach and learn from microscopic images. Our long-term hope is that virtual slide technology will be promoted nationwide in the numerous innovative areas as those described above, so that it becomes an efficient and affordable technology for all. As teachers, using virtual microscopy we can get more information about how our students learn pathology, how instructive the laboratory practicals are and which slides are of real didactic value for the students.

## Competing interests

The authors declare that they have no competing interests.

**Figure 1:**
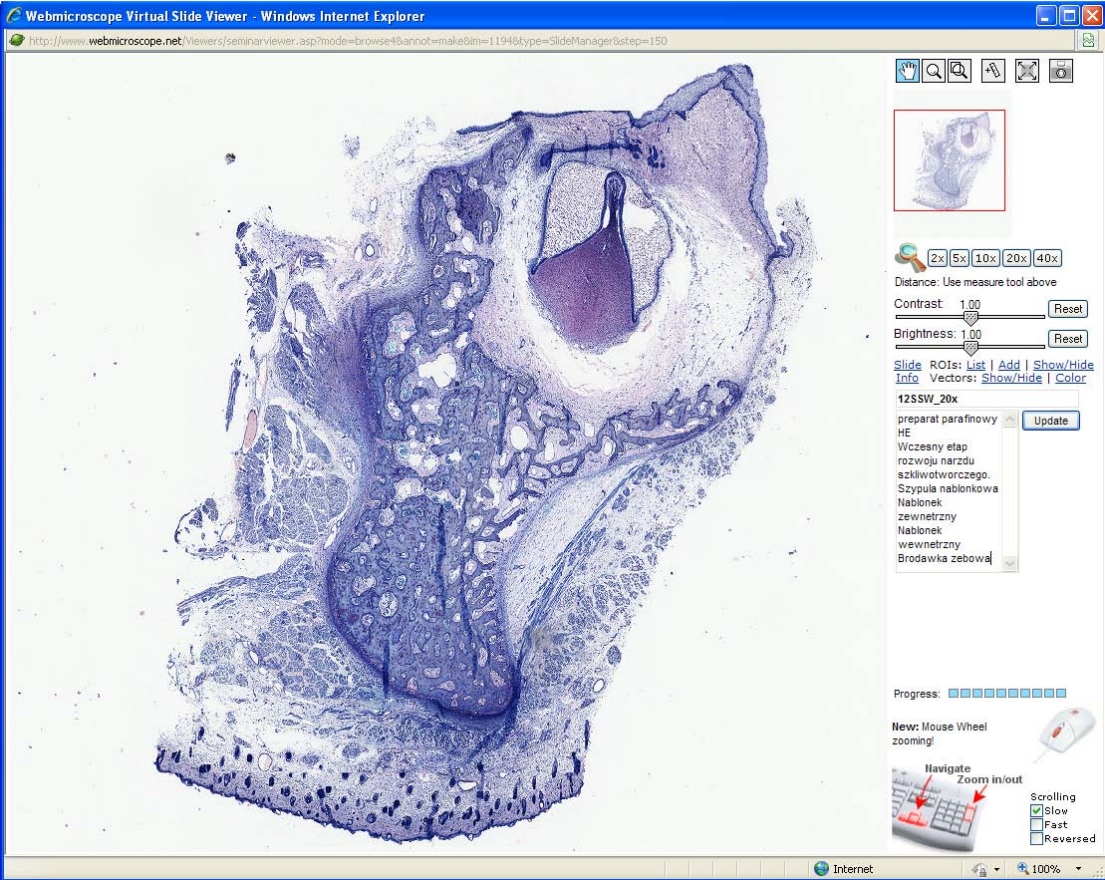
**Screenshot of WebMicroscope plug-in viewer.** This is screen shot of the WebMicroscope for the oral pathology course. Using the navigational tools at the top of the right frame, student can manipulate the virtual slide through five levels of magnification, starting at the level of the whole mount, as well as click and drag the slide in an x-y axis through the entire surface of the slide, at any magnification. In the lower frame is the text of the laboratory syllabus. A view of early bell stage of odontogenesis is illustrated.
